# Postprandial Effects of a Proprietary Milk Protein Hydrolysate Containing Bioactive Peptides in Prediabetic Subjects

**DOI:** 10.3390/nu11071700

**Published:** 2019-07-23

**Authors:** Tina Sartorius, Andrea Weidner, Tanita Dharsono, Audrey Boulier, Manfred Wilhelm, Christiane Schön

**Affiliations:** 1 BioTeSys GmbH, Schelztorstr. 54–56, 73728 Esslingen, Germany; 2 Ingredia S.A., 51 Avenue F. Lobbedez CS 60946, 62033 Arras CEDEX, France; 3 Department of Mathematics, Natural and Economic Sciences, Ulm University of Applied Sciences, Albert-Einstein-Allee 55, 89081 Ulm, Germany

**Keywords:** alpha-glucosidase inhibitor, biopeptides, blood glucose, glycemic control, hyperglycemia, milk peptides, postprandial, prediabetes, pre-meal, type 2 diabetes

## Abstract

Milk proteins have been hypothesized to protect against type 2 diabetes (T2DM) by beneficially modulating glycemic response, predominantly in the postprandial status. This potential is, amongst others, attributed to the high content of whey proteins, which are commonly a product of cheese production. However, native whey has received substantial attention due to its higher leucine content, and its postprandial glycemic effect has not been assessed thus far in prediabetes. In the present study, the impact of a milk protein hydrolysate of native whey origin with alpha-glucosidase inhibiting properties was determined in prediabetics in a randomized, cross-over trial. Subjects received a single dose of placebo or low- or high-dosed milk protein hydrolysate prior to a challenge meal high in carbohydrates. Concentration–time curves of glucose and insulin were assessed. Incremental areas under the curve (iAUC) of glucose as the primary outcome were significantly reduced by low-dosed milk peptides compared to placebo (*p* = 0.0472), and a minor insulinotropic effect was seen. A longer intervention period with the low-dosed product did not strengthen glucose response but significantly reduced HbA_1c_ values (*p* = 0.0244). In conclusion, the current milk protein hydrolysate of native whey origin has the potential to modulate postprandial hyperglycemia and hence may contribute in reducing the future risk of developing T2DM.

## 1. Introduction

Insulin resistance, a condition established by genetic and environmental factors, leads to impaired glucose tolerance due to an imbalance between insulin sensitivity and insulin secretion. This so-called prediabetic status plays an important pathophysiological role in the development of type 2 diabetes mellitus (T2DM) and is a hallmark of obesity, dyslipidemias, and other major risk factors contributing to the metabolic syndrome [1]. Over the years, our understanding of insulin resistance has improved tremendously, while T2DM is expected to have increasing detrimental effects on the health of populations and healthcare systems. Aside from preventive activities, to combat sedentary lifestyles and unbalanced diets in particular, reducing postprandial glycemia is as important as lowering fasting blood glucose levels to limit (or at least delay) the appearance of T2DM in at-risk individuals, and even modest postprandial hyperglycemia may lead to β-cell dysfunction [2,3]. As such, numerous studies have consistently demonstrated that pathophysiological abnormalities associated with an increased postprandial hyperglycemia ≥155 mg/dL (value of 1 h postload glucose concentration) including impaired insulin sensitivity, β-cell dysfunction, and increased glucose intestinal absorption, which are linked to an increased risk for future T2DM [2]. Most anti-diabetic agents that are currently available reduce fasting blood glucose levels but have little impact on postprandial glycemic excursions and thus do not normalize postprandial hyperglycemia [4]. In this context, simple dietary modifications, proper nutrition, and exercise may modify postprandial derailments; also, there is growing interest in food components that may beneficially modulate glycemic response, predominantly in the postprandial status.

There are many scientific reports highlighting the role of biologically active peptides derived from food proteins (e.g., milk, eggs, plant proteins), and clinical studies revealed that protein-rich dairy products are beneficial for reducing the risk of developing T2DM due to their glycemic and insulinotropic effect to improve glycemic status [5,6,7,8]. Thereby, the possible protective mechanism has been ascribed to the protein fraction [9,10,11]. Such biologically active protein fragments are released from parent proteins after enzymatic action (e.g., protein hydrolysis in the digestive tract) and positively influence various functions of the human body by interacting with enzymes or receptors [12,13]. Moreover, it is assumed that milk proteins have more effects on metabolic response in subjects with disturbed glucose metabolism [14]. Milk comprises two protein fractions, the slowly digestible casein and the fast digestible whey fraction [15]. Whey from native origin is produced by direct filtration of pasteurized skimmed milk and is therefore a more native protein compared to whey protein from cheese production with more denaturated protein character and less leucine content than native whey [16]. A further degradation of proteins is achieved by hydrolysis in mainly di- and tri-peptides via proteolytic enzymes in the digestive tract, resulting in a complex mixture of peptides of different length and free amino acids [17]. The hydrolysis process may change the kinetic pattern of the specific protein fractions, as shown for casein with more rapid digestion characteristics [15,18,19] and for whey with an improved absorption in a perfused human jejunum model [20]. Milk-derived bioactive peptides can be encrypted in both casein (α-, β-, and γ-casein) and whey proteins (β-lactoglobulin, α-lactalbumin, serum albumin, immunoglobulins, lactoferrin, and protease-peptone fractions) [21]. Also, milk protein hydrolysates were previously analyzed for their antidiabetic properties due to inhibition of alpha-glucosidase, a carbohydrate degrading digestive enzyme [22,23]. Counteracting alpha-glucosidase action through inhibition delays carbohydrate hydrolysis and consequently extends its digestion time (gastric emptying), which results in reduced glucose absorption from the gastrointestinal tract. Thus, postprandial levels of blood glucose and insulin are reduced [23,24]. The alpha-glucosidase inhibitor acarbose was shown to be an effective and valuable option in delaying or preventing the progression to T2DM [25]. However, this oral antidiabetic drug is known to cause gastrointestinal side effects when used as long-term therapy. Thus, in the last few years, progress has been attained in the search for new peptide alpha-glucosidase inhibitors, either synthetic or of natural origin.

The purpose of our study was to evaluate whether a proprietary milk protein hydrolysate of native whey origin containing alpha-glucosidase inhibiting bioactive peptides might improve postprandial glucose profile after single dosage or after a six week intervention period in prediabetic subjects. Therefore, both glucose response and insulin secretion were assessed from individual concentration–time curves. We used Pep2Dia^®^ as an investigational product containing a bioactive arginine-proline (AP) dipeptide with alpha-glucosidase inhibiting properties. Based on literature and on proprietary in vitro studies according to Kang et al. [26] (European Patent EP 3,107,556), there is evidence that the current milk protein hydrolysate acts on the inhibition of alpha-glucosidase with an IC_50_ value of 0.0025 mg/mL and thereby reduces glucose absorption from the gastrointestinal tract [27].

## 2. Materials and Methods

### 2.1. Study Subjects

**Figure 1:**
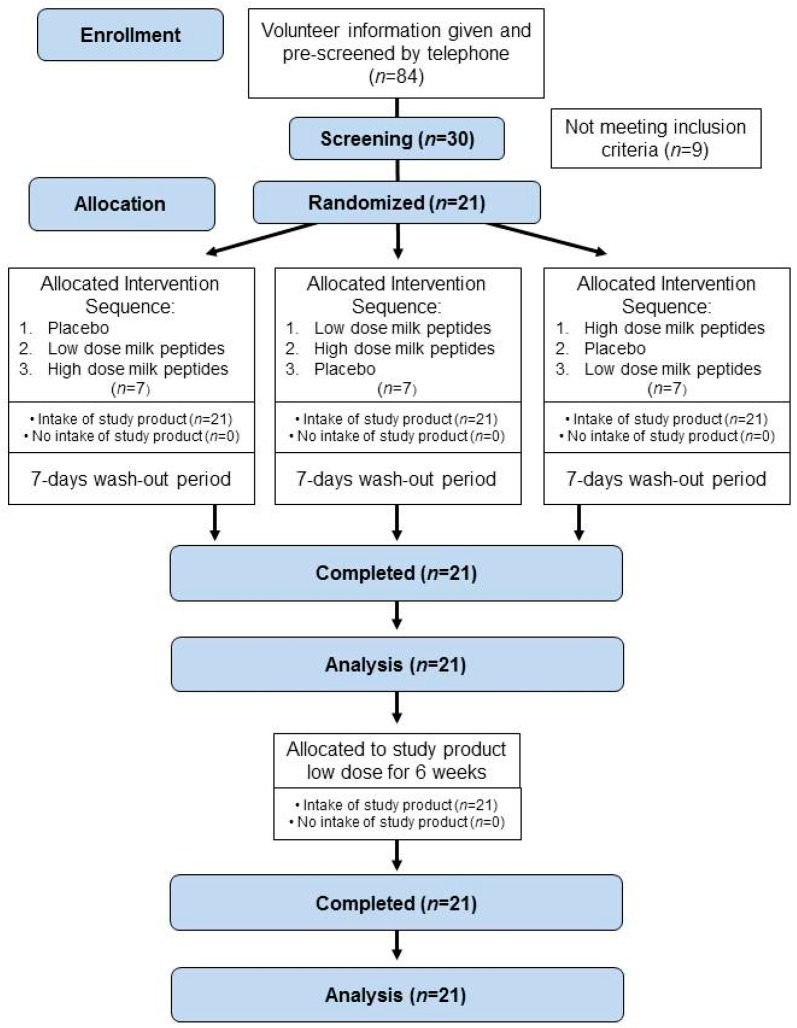
Subject recruitment flow chart.

From September 2018 to January 2019, a total of 21 subjects were included in the monocentric study at BioTeSys GmbH (Esslingen am Neckar, Germany). Overall, 84 non-smoking female and male people aged 30–70 years with a body mass index (BMI) of 19–35 kg/m^2^ were pre-screened for eligibility, from which 30 subjects were screened to ascertain their eligibility. The main inclusion criteria were prediabetic HbA_1c_ values between 5.7% to 6.4% and/or fasting glucose ≥5.6 mmol/L (≥100 mg/dL) and <7.0 mmol/L (<125 mg/dL), confirmed twice on two separate days if HbA_1c_ value was <5.7%. The subjects had to be in good physical and mental health represented by the medical history, physical examination, electrocardiogram, vital signs, and results of biochemistry and hematology. Finally, 21 subjects (8 men, 13 women) were included and all completed the study successfully, as shown in Figure 1.

The main exclusion criteria were a relevant history or presence of any medical disorder potentially interfering with this study (e.g., malabsorption, chronic gastro-intestinal diseases, severe depression, cardiovascular disease occurrence within the last 3 months, etc.), regular intake of medications or supplements known to affect glucose tolerance, diagnosed type 2 diabetics with medical treatment, and drug, alcohol, and medication abuses. Medications for treatment of chronic diseases that do not affect the metabolism of the study product were permitted and were judged individually regarding interference with the study by an investigator. Any concomitant chronic disease medication and medication used for the treatment of adverse events (AEs) was documented.

With regard to the 84 subjects given information and pre-screened by phone interview, the inaptitude was due to fasting glucose levels ≤100 mg/dL or >125 mg/dL in previous laboratory reports from subjects’ physician, BMI >35 kg/m^2^, metformin medication, lack of interest/response, and time collision.

This study was conducted in orientation towards the guidelines of the Declaration of Helsinki and Good Clinical Practice. The protocol and all documents were approved by the Institutional Review Board (IRB) of Landesärztekammer Baden-Württemberg with the reference number F-2018-062. A written informed Consent Form was obtained from all participants prior to screening evaluations. The present study was registered with ClinicalTrials.gov (ID: NCT03,932,695).

### 2.2. Study Design

The study was performed as a randomized, double-blind, placebo-controlled, monocentric, 3-way-cross-over study with 21 eligible subjects under fasted conditions at the study site of BioTeSys GmbH, Esslingen, Germany. A CONSORT 2010 checklist of information is included in the Supplementary Materials (Table S1). There was a wash-out period of 7 days between the study days to assess postprandial glucose response after a challenge meal. Within the cross-over study design, subjects received all interventions randomly allocated to 3 sequence groups.

Following an overnight fasting period of at least 10 h, a permanent venous catheter was inserted, and baseline blood (time points were 10 min and 5 min prior to challenge meal) was examined at the three visits within the cross-over study (kinetic days). Subjects received a single dose of placebo or 1400 mg (low dose) or 2800 mg (high dose) bioactive peptides from milk protein hydrolysate 15 min prior to a challenge meal high in carbohydrates (consisting of white bread, jam, and butter standardized to 75 g carbohydrates), and blood was further sampled at 15, 30, 45, 60, 90, 120, 150, and 180 min after the intake of the challenge meal. All participants received the two dosages of the study product and the placebo, and effects were compared to the placebo.

Additionally, an open-label single arm phase was performed with a daily intake of the low dose milk peptide concentration for 6 weeks to estimate effects over a longer period. After the 6 week intervention period, the postprandial assessment after the intake of 1400 mg bioactive peptides from milk protein hydrolysate 15 min prior to a challenge meal was repeated comparable to the cross-over phase. Subjects were encouraged not to change their food habits and physical activity during the study. Therefore, nutrition habit questionnaires were filled in during screening after the single dose cross-over study (= before 6 week intervention) and after 6 week intervention within the open-label single arm design. Thereby, subjects were asked about their food habits using a semi-quantitative short questionnaire assessing different food categories (fruit, vegetables, sausage, meat, intake of dairy products, sweets including beverages).

Blood analysis comprised determination of glucose and insulin plasma concentration over time at defined intervals besides blood routine parameters such as hematogram or total cholesterol. Subjects were asked to avoid alcohol 24 h before each study visit and to consume standardized meals 24 h prior to each visit to control for external confounding factors. In detail, breakfast was individually standardized, and for lunch, tortellini with pesto was served. Furthermore, a standardized snack (apple and cookie) was provided, and bread with cream cheese “Frischkäse” and cucumber had to be consumed as dinner. Additionally, subjects were not allowed to consume food or drink anything other than water for at least 10 h before testing, and no strenuous physical activity or endurance sports were allowed within 24 h before the study visits. In the morning of the study visits, subjects were instructed to drink a minimum of 200 mL water after waking up before they came to the study site.

### 2.3. Intervention

The investigational product (Pep2Dia^®^) was a milk protein hydrolysate from native whey protein containing a bioactive arginine-proline (AP) dipeptide (between 0.15% and 0.4%) with alpha-glucosidase inhibiting properties. The proprietary compound was prepared by Ingredia S.A. (Arras CEDEX, France) and was produced from native whey extracted by filtration according to Boutrou et al. [28]. Furthermore, a protease was used to perform the respective procedure. The protein is composed of 100% soluble protein with mainly β-lactoglobulin and α-lactalbumin. The profile of the peptides in the investigational product was as follows: 94.5% with a molecular weight (MW) <5000 Da, 0.5% with 5000–10,000 Da MW, and 5% with a MW > 10,000 Da. The products were provided in capsules (vegetable fiber) with 350 mg of milk protein hydrolysate per capsule (which includes, on average, 0.96 mg AP peptide). Maltodextrin with dextrose equivalent of 9 (DE9) was used as placebo with 350 mg per capsule. For single dose intake, 4 verum capsules and 4 placebo capsules (low dose) or, for high dose, 8 verum capsules were taken 15 min prior to a challenge meal. In the open-label single arm phase, subjects consumed the investigational product (4 verum capsules) daily 15 min prior to lunch, and at the study visit, subjects ingested 4 verum capsules after an overnight fasting period 15 min prior to a challenge meal. Manufacturing and encapsulation were carried out in compliance with Good Manufacturing Practice conditions, and all excipients as well as capsule shells met the current European food regulations. Size, shape, color, odor, and secondary packaging were identical between verum and placebo capsules to ensure double-blind conditions. Capsules were provided by Ingredia S.A. (Arras CEDEX, France), and subjects received either placebo or low dose (1400 mg) or high dose (2800 mg) milk protein hydrolysate.

### 2.4. Sample Collection and Processing

Venous blood samples were taken at screening visits to assess safety parameters (differentiated hematogram and clinical laboratory). At the same day, analyses with standard methods were performed at an accredited laboratory (Synlab Medizinisches Versorgungszentrum Leinfelden-Echterdingen, Germany). Blood samples were centrifuged at 3000× *g* for 10 min at 4 °C, and aliquots for spare samples for the determination of glucose and insulin were taken. Plasma glucose was analyzed using the Atellica^®^ CH analyzer (Siemens Healthcare GmbH, Germany; assay: Atellica CH Glucose Hexokinase_3, Ref. 11,097,592) with enzymatic UV detection based on the glucose hexokinase method. Briefly, glucose-6-phosphate formed from glucose and ATP by hexokinase was oxidized by NAD^+^ in a reaction catalyzed by glucose-6-phosphate dehydrogenase to give NADH, which was quantitated spectrophotometrically at 340/410 nm. Serum insulin was analyzed using the Atellica^®^ IM analyzer (Siemens Healthcare GmbH, Germany; assay: Atellica IM IRI, Ref. 10,995,628) with insulin detection based on a sandwich-type of electrochemiluminescence immunoassay using two monoclonal antibodies against insulin. Thereby, insulin quantification was linked to the number of relative light units (RLUs). Fasting blood glucose was controlled in finger prick samples using the HemoCue Glucose 201+ Analyzer (HITADO GmbH, Möhnesee, Germany) on the morning of each study day.

### 2.5. Methods for Safety (Adverse Events, Concomitant Medication, and Tolerability)

During the study intervention, the subjects documented any adverse events and concomitant medication. The tolerability was assessed at the end of the study days. The subjects rated overall tolerability to three categories from “well tolerated”, “slightly unpleasant”, or “very unpleasant”.

### 2.6. Data Analysis and Statistics

Based on previous data [29] reporting a reduction of postprandial glucose levels after a challenge meal with different milk proteins with up to 18% reduction, a conservative assumption with a reduction of 11% was applied for the prior sample size calculation, resulting in an effect size of d = 0.74. Based on the following input details—alpha error problem of α = 0.05, actual power of 80%, correlation between groups of 0.5—a sample size of *n* = 17 subjects was estimated, which was applied for the 3-way cross-over design in phase I. Considering a drop-out rate of 15% and equally sized sequence groups for the 3-way cross-over design in phase I, the study was performed with *n* = 21 subjects. The part II open-label phase was planned to be exploratory as a first proof of concept study to estimate long-term effects and to gain first experiences for further clinical studies. Pharmacokinetic parameters were individually calculated with the blood concentration–time curves. As the primary efficacy endpoint, the incremental area under the observed concentration–time curve above the baseline (iAUC), more precisely iAUC_0–180 min_, was calculated by applying the trapezoidal rule with the y-axis, defined by glucose plasma concentration, and the x-axis defined via sampling time points. Secondary efficacy target variables were iAUC_0–180 min_ of insulin, total AUC_0–180 min_, and ΔC_max_ of glucose and insulin. Primary and secondary endpoints were analyzed using a linear mixed model of iAUC with treatment (3 levels), period (3 levels), sequence (3 levels), and baseline blood glucose level within study periods as fixed effects and subject as random effect. Due to the 7 days wash-out period, examination of possible carry-over effects was not foreseen. The residuals of this model were checked for normality using the Shapiro–Wilk test with an alpha level of 0.05. If applicable, data were log transformed prior to analysis. Multiple pairwise comparisons of least squares means of primary and secondary endpoints were adjusted by the method of Dunnett–Hsu in order to assess differences between the two active treatments and placebo. Data of the cross-over design are presented as least square means with 95% confidence interval (CI).

Moreover, in the open-label study period, besides HbA_1c_ values, the homeostasis model assessment (HOMA) index and the Matsuda index were used to evaluate the impact of the study product intake during a longer period on insulin sensitivity, and comparisons were performed between the baseline and the end of intervention. Additionally, during the open-label study period of 6 weeks, the pharmacokinetic endpoints after the challenge test were compared with placebo during the study phase I. Data were evaluated using a paired t-test. In case of non-normal distribution of data, a Wilcoxon signed-rank test was applied. Data of the open label phase are presented as means with 95% CI. All 21 subjects were included in the analysis. Statistical tests were performed two-sided, and *p* values < 0.05 were statistically significant. Statistical evaluation, summary tables, and graphs were generated using GraphPad Prism software (La Jolla, CA, USA) and SAS V9.4 statistical software (SAS Institute, Cary, North Carolina).

## 3. Results

### 3.1. Subject Characteristics

The investigated study population was a non-smoking prediabetic study group, on average 62.4 years (95% CI: 60.0–64.9) old with a BMI of 28.1 kg/m^2^ (95% CI: 26.3–30.0). A total of 21 subjects (*n* = 13 women, *n* = 8 men) completed the study.

Table 1 presents the participants’ demographic data and screening data. Vital signs and blood routine parameters were within normal range. None of the subjects were vegetarian or vegan, and 52% of the participants practiced sports on a regular basis.

**Figure 2:**
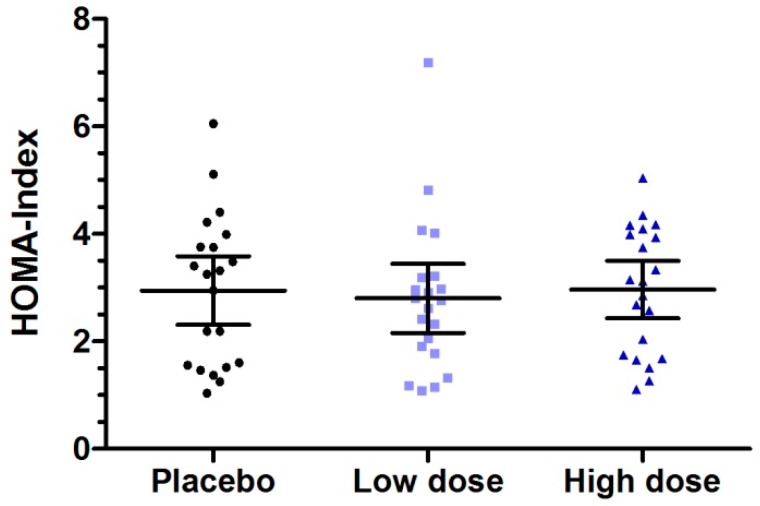
Distribution of homeostasis model assessment (HOMA) index at baseline at the testing days before intake of placebo or milk protein hydrolysate containing bioactive milk peptides in low and high doses. Data represent mean ±95% CI. Data represent mean ±95% CI. No statistical difference between baseline conditions.

Subjects were advised not to change their eating habits, which were controlled by a semi-quantitative nutrition habit questionnaire comprising 24 food categories. There was no significant change over intervention period (*p* = 0.2148). Fasting baseline values of glucose and insulin did not differ among the single dose treatment days (*p* > 0.05). Regarding the homeostasis model assessment of insulin resistance (HOMA-IR), a parameter that estimates insulin sensitivity considering the relation between fasting insulin and fasting glucose, was—on average—clearly above the cut-off level of two [30], indicating insulin resistance and not different among the testing days (placebo: 2.95; low dose: 2.80; high dose: 2.96; six week intervention: 2.84; Figure 2).

### 3.2. Milk Peptides and Their Postprandial Effect on Glucose Response after Single Dose Intake

**Figure 3:**
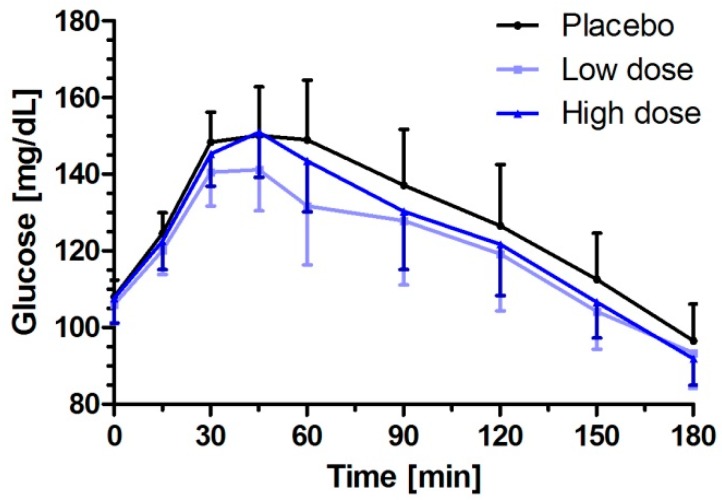
Glucose responses to a single intake of placebo or low- or high-dosed milk protein hydrolysate containing bioactive peptides consumed 15 min prior to a challenge meal. Data represent mean ±95% CI.

There was a significant increase of plasma glucose concentration over time after the challenge meal (*p* < 0.0001 after all study interventions). The concentration–time curves indicate that milk peptides have an impact on postprandial blood glucose profile in prediabetic subjects (Figure 3). No dose linearity between low dose (1400 mg) and high dose (2800 mg) milk peptides could be revealed, and the effects were even slightly more distinct after single dose intake of low dose milk peptides in comparison to the high dose.

In terms of iAUC_0–180 min_ glucose, single dose intake of low dose milk peptides resulted in significantly reduced values compared to the placebo (3441.1 vs. 4312.0 mg/dL × min, *p* = 0.0472), whereas the high dose milk peptides were not statistically different to the placebo (*p* = 0.1749) (Table 2). The secondary endpoint ∆C_max_, the maximum increase of glucose above baseline, confirmed the significant postprandial glucose lowering effect of the low dose milk peptides with a mean increase in plasma glucose of 44.8 mg/dL (95% CI: 35.9–53.8) vs. 52.8 mg/dL (95% CI: 43.9–61.8) for placebo (*p* = 0.0237) vs. 49.1 mg/dL (95% CI: 40.1–58.0) for high dose milk peptides (Table 2). In addition, analyses of total AUC_0–180 min_ and C_max_ revealed statistical significance for the low dose milk peptides in comparison to the placebo (low dose vs. placebo: AUC_0–180 min_: 21,931 vs. 23,073 mg/dL × min, *p* = 0.0313; C_max_: 152.1 mg/dL (95% CI: 143.1–161.0) vs. 160.1 mg/dL (95% CI: 151.1–169.0), *p* = 0.0237).

### 3.3. Milk Peptides and Their Postprandial Effect on Insulin Response after Single Dose Intake

**Figure 4:**
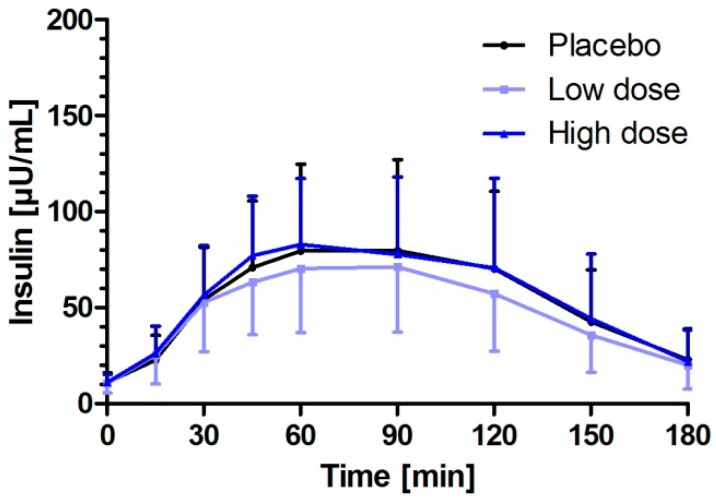
Insulin responses to low and high dose milk protein hydrolysate containing bioactive peptides consumed 15 min prior to a challenge meal. Data represent mean ±95% CI.

The impact of milk peptides on insulin release as a response to the challenge meal was a minor evident (Figure 4). There was a slight reduction of iAUC of insulin after an intake of low dose milk peptides in comparison to the placebo [low dose: 6339.8 µU/mL × min (95% CI: 4997.5–8042.5); placebo: 6844.5 µU/mL × min (95% CI: 5396.5–8681.9)]; however, the difference was not significant (*p* = 0.4296). No difference to the placebo was seen in the high dose milk peptides (7212.0 µU/mL × min (95% CI: 5396.5–8681.9) vs. 6844.5 µU/mL × min (95% CI: 5396.5–8681.9), *p* = 0.6606). The maximum increase in plasma insulin (∆C_max_) after the challenge meal was lower for low dose milk peptides compared to the placebo and the high dose milk peptides [low dose: 66.2 µU/mL (95% CI: 54.8–80.1); high dose: 74.1 µU/mL (95% CI: 61.3–89.5); placebo: 71.4 µU/mL (95% CI: 59.0–86.3)]. ∆C_max_ of both low and high dose milk peptides were not different to the placebo (low dose: *p* = 0.5536; high dose: *p* = 0.8573). These results indicate a negligible insulinotropic effect of the current milk protein hydrolysate.

### 3.4. Six Week Intervention with Low Dose Milk Peptides

**Figure 5:**
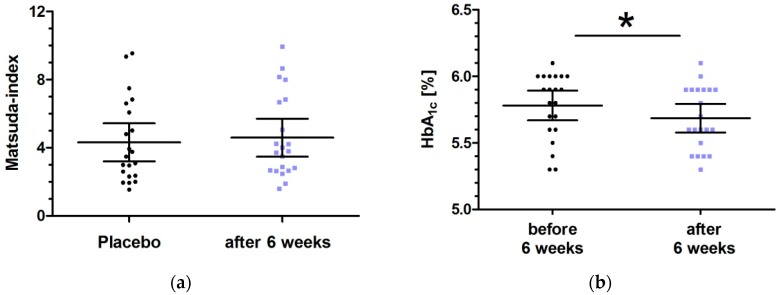
Distribution of Matsuda index (**a**) and of HbA_1c_ [%] values (**b**) after the six week intervention period with low dose milk protein hydrolysate containing bioactive milk peptides. Data represent mean ±95% CI. Statistical difference as indicated: * *p* < 0.05.

Plasma concentrations of fasting blood glucose and fasting insulin after six weeks of low dose milk peptides intervention were comparable with the fasting conditions prior to the challenge meal with single dose placebo intervention [baseline vs. six week intervention: 108.0 mg/dL (95% CI: 103.6–112.4) vs. 106.8 mg/dL (95% CI: 102.4–111.1) for glucose (*p* = 0.5165); 11.01 mg/dL (95% CI: 8.68–13.34) vs. 10.67 µU/mL (95% CI: 8.22–13.12) for insulin (*p* = 0.3352)]. Approximation of whole-body insulin sensitivity, which combines both hepatic and peripheral tissue insulin sensitivity, was performed by assessment of the Matsuda index; 61.9% of subjects were in the pathological range with values <4, 9.5% in the borderline range with values between 4 and 6, and 28.6% were in the normal (healthy) range with values of 6–12 at baseline, defined as the condition prior to the six week intervention period. Daily intake of low dose milk peptides for six weeks did not result in a change of HOMA-IR (baseline vs. six week intervention: 2.87 (95% CI: 2.28–3.45) vs. 2.84 (95% CI 2.14–3.53); *p* = 0.5202)), but resulted in a slight increase of the Matsuda index by trend [baseline vs. six week intervention: 4.32 (95% CI: 3.21–5.43) vs. 4.59 (95% CI: 3.48–5.71); *p* = 0.0952)] (Figure 5a). There was a significant reduction of HbA_1c_ levels after a six week intervention treatment with low dose milk peptides resulting in HbA_1c_ values of 5.69% (95% CI: 5.58–5.79) compared to baseline values of 5.78% (95% CI: 5.67–5.89) with *p* = 0.0244 (Figure 5b). Notably, 11 out of 21 subjects (52.4%) completed with HbA_1c_ levels <5.7% after the six week intervention period.

**Figure 6:**
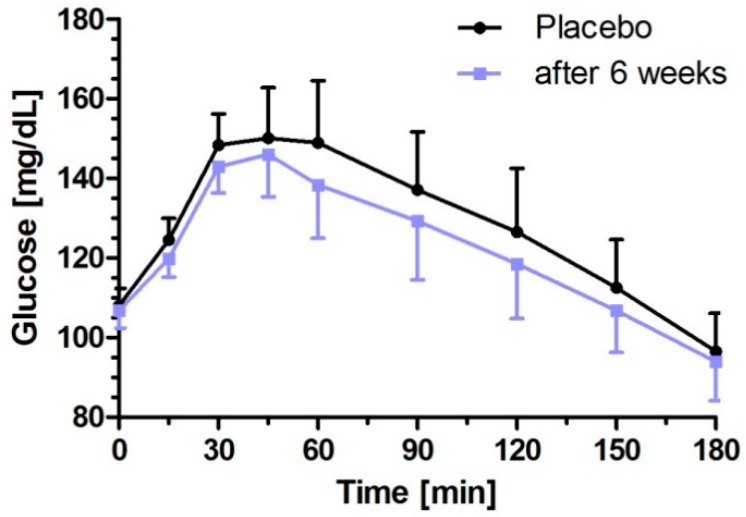
Glucose responses after six week intervention with low dose milk protein hydrolysate containing bioactive milk peptides and after challenge meal. Data represent mean ±95% CI.

In accordance with the single dose treatment, the concentration–time curve of postprandial plasma glucose concentration in response to the challenge meal after six week intervention with low dose milk peptides was below the placebo intervention at all time points (0–180 min) (Figure 6). However, the six week intervention period did not strengthen the acute postprandial glucose response in comparison with the single dose intake of low dose milk peptides, as iAUC values of glucose were similar [single dose vs. six week intervention: 3423 mg/dL × min (95% CI: 2181–4664) vs. 3577 mg/dL × min (95% CI: 2305–4849); *p* = 0.6766)] but statistically different to placebo (*p* = 0.037).

Moreover, glucose response analyses in terms of ∆C_max_ and total AUC_0–180 min_ supported the abovementioned primary endpoints and confirmed the significant postprandial glucose lowering effects after low dose milk peptides intervention over a longer period of six weeks (placebo vs. six week intervention: 53.1 mg/dL (95% CI: 44.1–62.1) vs. 47.5 mg/dL (95% CI: 39.2–55.9), *p* = 0.0399 for ∆C_max_, and 23,211 mg/dL × min (95% CI: 21,280–25,143) vs. 22,099 mg/dL × min (95% CI: 20,393–23,804); *p* = 0.0408 for AUC_0–180 min_)).

Low dose milk peptides intervention over a period of six weeks had no impact on the insulin response compared to the single dose intake regarding iAUC and ∆C_max_. Again, although descriptively, there was (on average) a slight reduction by trend of iAUC of insulin in comparison to the placebo with *p* = 0.0952 for iAUC insulin (after six weeks: 7434 µU/mL × min (95% CI: 5770–9097); placebo: 8163 µU/mL × min (95% CI: 5962–10,363)). This was confirmed by ∆C_max_ values of insulin (after 6 weeks: 76.7 µU/mL (95% CI: 61.4–92.1); placebo: 80.4 µU/mL (95% CI: 61.7–99.1), *p* = 0.3048)).

### 3.5. Safety Assessment

All subjects (100%) rated the tolerability of the study products as “well tolerated” during the kinetic days of single dose intake and after the six week intervention period with low dose milk peptides. During the assessment of postprandial glucose response after a challenge meal after single dose intake, no adverse events (AEs) were reported. In terms of the six week intervention period with low dose milk peptides, a total of 14 adverse events were assessed by 10 subjects (predominantly headaches (6 x) and common cold (5 x)). Of those AEs, one serious adverse event (SAE) was reported on one surgery accompanied with hospitalization. None of the AEs were related to the study product.

## 4. Discussion

In the present study, we investigated the impact of a proprietary milk protein hydrolysate from native whey origin containing a bioactive AP dipeptide on postprandial glucose and insulin responses after a challenge meal in prediabetic subjects after a single dosage regimen or over a longer period of six weeks. Based on literature and on proprietary in vitro studies according to Kang et al. [26] (European Patent EP 3107,556), there is evidence that the current milk protein hydrolysate containing bioactive AP dipeptides acts on the inhibition of alpha-glucosidase with an IC_50_ value of 0.0025 mg/mL and thereby reduces glucose absorption from the gastrointestinal tract [27]. The amount of the bioactive AP dipeptide per capsule is, on average, 0.96 mg and thus in line with the content of already published bioactive peptides of whey protein origin [31], irrespective of the metabolic effects. The concentration–time curves indicated that the study product has the potential to counteract postprandial hyperglycemia in prediabetic subjects. After single dose application, effects on glucose response were slightly more distinct by intake of low-dosed milk peptides (1400 mg) 15 min prior to a challenge meal in comparison to the high dose (2800 mg) in terms of reduced iAUC glucose. Compared to placebo, a significant difference was seen for the low dosage (*p* = 0.0472) but not for the high dose. Of note, no linear dose–response relationship could be revealed. This might have been due to the multi-peptide characteristics, and interactions of single components in different concentrations might have been responsible for the limited dose–response. However, this needs further exploration in future studies. In addition, the secondary endpoints ΔC_max_, AUC_0–180 min,_ and C_max_ supported the findings for the primary endpoint iAUC and confirmed the significant postprandial glucose lowering effects after single dose intake of the low-dosed milk peptide.

Milk protein hydrolysates were previously analyzed for their antidiabetic properties with an alpha-glucosidase inhibiting effect [22,23]. Thereby, potent peptide fractions of a whey protein concentrate were identified with high biological activities of peptide fractions with a molecular weight lower than 33 kDa [32]. In what way the biological activity due to molecular weight might be causative for the postprandial glucose response exceeds the objective of the current study. Compared to already published literature in which the pre-meal effect of milk proteins (whey proteins) were analyzed in subjects with and without T2DM, an absent glucose response was demonstrated in both groups [14] owing to the insulinotropic rather than the glycemic effect of whey protein, which has higher amounts of lysine, threonine, tryptophan, leucine, and isoleucine [33]. Of note, recent in vitro data using preadipocytes revealed that the tripeptides IPP (Ile-Pro-Pro) and VPP (Val-Pro-Pro), which are derived from milk casein, enhance insulin sensitivity and contribute toward the prevention of insulin resistance in the presence of tumor necrosis factor [34]. VPP-mediated improved insulin sensitivity was also confirmed in diet-induced obese mice by decreasing pro-inflammatory cytokines in adipose tissue [35]. Further, it is known that whey and casein proteins differentially affect postprandial glucose and insulin response. It was shown that insulin secretion was greater with whey protein than with casein, whereas incretin responses in terms of GLP-1 tended to be lower with casein than with whey protein [36].

Analysis of iAUC of insulin release of the individual concentration–time curves revealed that the study product’s impact on insulin release was minor, evident from the response to the challenge meal. However, after single dose application there was, although descriptively, on average a slight reduction of iAUC of insulin after intake of the low-dosed milk peptide in comparison to the placebo but without reaching statistical significance.

One has to take into account that differential patterns in insulin response after milk protein intake were reported between studies, which may be the result of a number of fundamental differences in study design, such as preload design and the type of milk proteins, protein amount, or altered milk peptides/bioactive peptide sequences. Results of our study contrast with previous literature demonstrating a significantly reduced glucose response with a concomitant increase in insulin AUC by intake of 18 g milk protein (whey) to 25 g glucose [37]. This effect was ascribed to amino acid availability, which may potentiate the increased insulin response since plasma amino acids also increased in a dose-dependent manner. Similarly, a combination of whey and free amino acids induced a rapid insulinotropic effect, which influenced early glycemia [38]. Further literature demonstrated an insulinotropic effect of milk proteins or whey protein in terms of higher insulin release [14,39,40]. It is discussed that the insulinotropic properties appear to originate from a specific postprandial plasma amino acid pattern with predominantly isoleucine, leucine, lysine, threonine, and valine, the main amino acids of whey protein [38]. Whether the difference in the respective glycemic and/or the insulinotropic responses of the current milk protein hydrolysate with bioactive peptides might be related to a different incretin pattern or to changes in plasma amino acid concentration was not clarified in the present study. However, one has to mention that the current milk protein hydrolysate is of native whey origin, the cleanest and the least processed whey protein available, whereas most of clinical trials used regular whey protein from cheese whey (e.g., [14,33,37,38,41]). Due to the process of creating native whey, namely filtration of pasteurized skimmed milk, more proteins remain intact and thus there is a higher leucine content than the more common whey protein concentrate from cheese production [42]. Of note, it has been shown that intake of native whey protein induces greater leucine blood concentrations than other whey protein supplements [16]. Whether the higher leucine content of the native whey protein might be causative for the more glycemic than insulinotropic response after the challenge meal is speculative but might be an explanation to already published data from other groups using regular whey protein from cheese whey.

In addition, it may be considered that a glycemic response does not necessarily impact insulin release. In this context, the inconsistency between glycemic and insulinotropic responses to fresh milk and two fermented milk products in healthy subjects was previously addressed [39]. However, it is known that whey protein in particular tends to be less glycemic and more insulinotropic [40], and casein, another bioactive milk component, was reported to reduce the postprandial rise in blood glucose by an increased insulin response and blood glucose disposal in T2DM subjects when coingested with carbohydrates [43,44,45]. Interestingly, one study assessed the glycemic response following consumption of liquid protein preloads of whey (55 g) and casein (55 g) in comparison with lactose (56 g) and glucose (56 g) controls in overweight, prediabetic subjects [41]. Although a significant reduction in glucose response was shown, insulin concentrations were not affected. Furthermore, no impact on post-meal insulinaemia in accordance with a 16% reduction in post-meal glycemia over 360 min in overweight subjects further supports observations of the current study product [46]. Thus, one might assume that the current milk protein hydrolysate containing bioactive peptides may influence plasma glucose via insulin-independent mechanisms. This is supported by in vitro experiments demonstrating the alpha-glucosidase acting mode of action for the study product (unpublished data). Therefore, one might speculate that the slight reduction in insulin release might be a secondary response due to lower postprandial increase of glucose.

We further assessed the effect of the milk protein hydrolysate with bioactive peptides for a longer period of six weeks with a daily intake of 1400 mg of the study product. Notably, this intervention resulted in a slight improvement of whole-body insulin sensitivity (hepatic and peripheral tissue insulin sensitivity) as assessed by the Matsuda index. The change was not significant (*p* = 0.0952), which might be attributed to the limited samples size and needs further confirmation in future studies. Additionally, one might assume that the current milk protein hydrolysate containing bioactive peptides may influence whole-body insulin sensitivity secondary to its primary effects on alpha-glucosidase inhibition, which were not obvious after the limited intervention period of six weeks. In addition, the longer intervention period did not strengthen the postprandial effect on glucose response, as iAUC values were comparable to those of single dose intake.

In summary, the study product primarily influenced postprandial glycemia and secondarily influenced insulin sensitivity in the whole body, suggesting rather insulin-independent mechanisms or temporal changes in insulin sensitivity. Moreover, the six week intervention period accentuates the more glycemic and less insulinotropic effect of the current milk protein hydrolysate, as the glycemic marker HbA_1c_ was significantly reduced (*p* = 0.0244). Notably, 52.4% of the subjects completed the study with HbA_1c_ levels < 5.7% after the six week intervention period, and the significant reduction of HbA_1c_ is worth mentioning in the short time period of six weeks, which has to be confirmed in further studies with longer intervention periods.

Regarding study limitations, the current study was performed in cross-over design to control for inter-individual variability. This variability cannot be estimated from the data, as study products were only provided once to subjects. Nevertheless, data from the open-label single arm phase performed with a daily intake of the low-dosed milk peptide concentration for six weeks suggest minor inter-individual variability and overall confirmed the results of the three-way-cross-over study with single dose intake regimen. Furthermore, one has to take into account that T2DM—and even the prediabetic state—is a heterogeneous disease with multiple pathophysiologies. Both incretins and microbiota in the gastrointestinal tract are known to be affected in prediabetics [47,48], which might have an impact on the postprandial responses. Although these parameters were not assessed in this study, the current results look very promising and should be confirmed in further investigations.

## 5. Conclusions

The objective of the current study was to assess whether alpha-glucosidase inhibiting bioactive peptides from milk protein hydrolysate might improve postprandial glucose profiles in prediabetic subjects. We demonstrated that low dose milk peptides had a significant impact on postprandial blood glucose profile with more glycemic than insulinotropic properties in prediabetic subjects after a challenge meal high in carbohydrates. This was confirmed after a single dose intake and after a six week intervention period, whereas impacts on postprandial effects were not strengthened by intervention over a longer period. Furthermore, the study product primarily influenced postprandial glycemia and secondarily influenced insulin sensitivity in the whole body, as only a minor increase of the Matsuda index and a slight but significant reduction of HbA_1C_ levels were demonstrated after the six week intervention period.

The investigated hydrolyzed milk-derived bioactive peptides (1.4 g/day) of native whey origin seem to be promising and well-tolerated by prediabetic subjects to control postprandial glucose levels, which should be confirmed in further clinical studies with longer intervention periods to ascertain the benefits for glucose homeostasis.

## Figures and Tables

**Table 1:** Demographic and screening data.

Variable	Prediabetics (*n* = 21)	
	Mean	95% CI
Age (years)	62.4	(60.0–64.9)
BMI (kg/m^2^)	28.1	(26.3–30.0)
Systolic BP (mmHg)	134.7	(127.3–142.1)
Diastolic BP (mmHg)	83.9	(79.6–88.2)
HbA_1c_ (%)	5.83	(5.69–5.97)
Fasting plasma glucose (mg/dL)	109.6	(105.3–113.9)

BMI: body mass index; BP: blood pressure; HbA_1c_: glycated haemoglobin.

**Table 2:** Postprandial glucose incremental areas under the curve (iAUC) and ∆C_max_ of glucose after single dose intake of placebo or low-or high-dosed milk protein hydrolysate. Group means referred to least squares (LS) means.

Glucose	Placebo	Low-Dosed Milk Protein Hydrolysate			High-Dosed Milk Protein Hydrolysate		
Variable	LS Mean 95% CI	LS Mean 95% CI	Treatment Difference 95% CI	*p*	LS Mean 95% CI	Treatment Difference 95% CI	*p*
iAUC_0–180 min_ (mg/dL × min)	4312.0 (2938.4–5685.6)	3441.1 (2066.9–4815.3)	−870.9 (−1732.4–−9.5)	0.0472	3685.8 (2312.6–5059.0)	−626.2 (−1480.5–228.1)	0.1749
∆C_max_ (mg/dL)	52.8 (43.9–61.8)	44.8 (35.9–53.8)	−8.00 (−15.02–−0.98)	0.0237	49.1 (40.1–58.0)	−3.78 (−10.75–3.19)	0.3627

## Supplementary Materials

The following are available online at https://www.mdpi.com/2072-6643/11/7/1700/s1, Table S1: CONSORT 2010 checklist of information to include when reporting a randomized trial.
