# Correction: Sartorius et al. “Postprandial Effects of a Proprietary Milk Protein Hydrolysate Containing Bioactive Peptides in Prediabetic Subjects” *Nutrients* 2019, *11*, 1700

**DOI:** 10.3390/nu12051266

**Published:** 2020-04-29

**Authors:** Tina Sartorius, Andrea Weidner, Tanita Dharsono, Audrey Boulier, Manfred Wilhelm, Christiane Schön

**Affiliations:** 1 BioTeSys GmbH, Schelztorstr. 54–56, 73728 Esslingen, Germany; t.sartorius@biotesys.de (T.S.); a.weidner@biotesys.de (A.W.); t.dharsono@biotesys.de (T.D.); 2 Ingredia S.A., 51 Avenue F. Lobbedez CS 60946, 62033 Arras CEDEX, France; a.boulier@ingredia.com; 3 Department of Mathematics, Natural and Economic Sciences, Ulm University of Applied Sciences, Albert-Einstein-Allee 55, 89081 Ulm, Germany; manfred.wilhelm@thu.de

**Keywords:** alpha-glucosidase inhibitor, biopeptides, blood glucose, glycemic control, hyperglycemia, milk peptides, postprandial, prediabetes, pre-meal, type 2 diabetes

## Abstract

Milk proteins have been hypothesized to protect against type 2 diabetes (T2DM) by beneficially modulating glycemic response, predominantly in the postprandial status. This potential is, amongst others, attributed to the high content of whey proteins, which are commonly a product of cheese production. However, native whey has received substantial attention due to its higher leucine content, and its postprandial glycemic effect has not been assessed thus far in prediabetes. In the present study, the impact of a milk protein hydrolysate of native whey origin with alpha-glucosidase inhibiting properties was determined in prediabetics in a randomized, cross-over trial. Subjects received a single dose of placebo or low- or high-dosed milk protein hydrolysate prior to a challenge meal high in carbohydrates. Concentration–time curves of glucose and insulin were assessed. Incremental areas under the curve (iAUC) of glucose as the primary outcome were significantly reduced by low-dosed milk peptides compared to placebo (p = 0.0472), and a minor insulinotropic effect was seen. A longer intervention period with the low-dosed product did not strengthen glucose response but significantly reduced HbA1c values (p = 0.0244). In conclusion, the current milk protein hydrolysate of native whey origin has the potential to modulate postprandial hyperglycemia and hence may contribute in reducing the future risk of developing T2DM.

The authors wish to make a correction to the published version of their paper [1].

Unfortunately, we noticed a mistake regarding the description of the bioactive dipeptide in our paper. Instead of “arginine–proline (AP)” the correct amino acid dipeptide is “alanine–proline (AP)”. In the published version of our paper [1], this affects two positions, namely:

1. Page 2 of 16: “We used Pep2Dia^®^ as an investigational product containing a bioactive alanine–proline (AP) dipeptide with alpha-glucosidase inhibiting properties”.
2. Page 4 of 16: “2.3 Intervention/ The investigational product (Pep2Dia^®^) was a milk protein hydrolysate from native whey protein containing a bioactive alanine–proline (AP) dipeptide (between 0.15% and 0.4%) with alpha-glucosidase inhibiting properties.”

The authors apologize to the readers for any inconvenience caused by the change. This change does not impact the content of the paper, the overall results or scientific conclusions. The original manuscript will remain online on the article webpage, with a reference to this correction.
