# Supplementary material for: Postprandial Effects of a Proprietary Milk Protein Hydrolysate Containing Bioactive Peptides in Prediabetic Subjects
Source: Nutrients. 2019 Jul 23;11(7):1700. doi: 10.3390/nu11071700 (PMC6683050; doi:10.3390/nu11071700)
Supplement: Supplementary file 1 [file nutrients-11-01700-s001.zip › nutrients-539701-Proof done supplementary.pdf]

CHOLÉ-DOC

Produits laitiers et prévention du diabète de type 2

Frédéric Fumeron
Inserm U695,
UFR de médecine de l'Université Paris Diderot, Paris

Il y a encore une décennie, la consommation de produits laitiers riches en matières grasses était considérée sans nuance par les épidémiologistes comme faisant partie du modèle alimentaire de type « Occidental », associée à un risque élevé de développer un diabète de type 2 [1]. Les graisses animales étaient tenues pour un facteur pathogène, sans tenir compte de la composition spécifique de la matière grasse laitière contenant de nombreux acides gras aux propriétés différentes, ni des autres composants du lait susceptible d'interagir avec celle-ci. Depuis une dizaine d'années, un certain nombre d'études épidémiologiques apportent des résultats susceptibles de remettre en cause cette pseudo-évidence. Une quinzaine d'études de cohortes longitudinales/prospectives ont été ainsi effectuées, dont l'étude française D.E.S.I.R., déjà détaillée dans ces colonnes [2; 3].

Epidémiologie

Le **tableau 1 (en annexe)** récapitule l'ensemble des études prospectives. Elles varient selon la durée du suivi et le nombre de cas incidents de diabète.

Une majorité d'études rapporte une association négative entre la consommation de produits laitiers totaux et/ou spécifiques et l'incidence du diabète de type 2. Aucune étude ne rapporte d'association positive. La plupart des études rapportant une association significative sont celles qui incluent le plus grand nombre de sujets. En d'autres termes, le diabète de type 2 étant une maladie d'origine multifactorielle, il est nécessaire d'étudier un grand nombre de sujets pour dégager une signification statistique. L'étude EPIC-Interact [4] est un cas particulier puisque dans cette étude multicentrique regroupant 8 pays d'Europe, la consommation totale de produits laitiers n'est pas associée à l'incidence du diabète de type 2. Cependant il existe une relation inverse entre la consommation de fromages et de produits fermentés (fromage, yaourt, lait fermenté) et l'incidence.

La deuxième étude des infirmières américaines, de type rétrospectif prospectif, renseigne sur les apports à l'adolescence [5]. Les femmes du quintile supérieur de consommation pour les produits entiers (2 portions/jour) ont un risque de diabète de type 2 diminué de 38% (RR=0,62 (0,47-0,83)). Après ajustement sur les facteurs de risque adultes, la relation demeure significative (RR=0,73 (0,54-0,97)). L'effet est très atténué après ajustement sur la consommation adulte, montrant que les bénéfices de la consommation adolescente sont liés à la persistance de la consommation. L'analyse conjointe des consommations adolescentes et adultes montre que les femmes avec des apports élevés de produits laitiers constants dans le temps sont protégées très significativement par rapport aux femmes à apports constamment faibles (RR=0,57 (0,39-0,82)).

Une méta-analyse toute récente portant sur 17 études prospectives totalisant 426 000 participants et près de 27 000 cas incidents de diabète montre un effet global significatif négatif entre la consommation de produits laitiers et l'incidence avec un RR moyen = 0,89 (IC=0,82-0,99) pour une consommation élevée *vs.* une faible consommation [6]. L'analyse dose-réponse montre un RR moyen de 0,93 (IC= 0,87-0,99) pour une consommation de 400 g/jour **(figure 1)**.

Lorsque les différentes catégories de produits laitiers sont examinées, les produits allégés, le fromage et les yaourts sont associés négativement au risque de diabète de type 2 **(tableau 2)**, mais selon les études on observe parfois des associations avec l'ensemble des produits fermentés ou les produits entiers. Pour le lait, on observe une tendance similaire (RR= 0,87 pour 200 g/jour), non significative sans doute en raison de la grande hétérogénéité des études. Cette méta-analyse met en évidence un aplatissement des courbes pour les consommations les plus élevées. En d'autres termes, augmenter la consommation au-delà de 400 g par jour pour les produits laitiers totaux ne semble pas apporter de bénéfices supplémentaires.

Une critique commune est que les effets attribués aux produits laitiers pourraient être dus

numéro
136
JUILLET - AOÛT
2013

www.cerin.org
CENTRE DE RECHERCHE ET D'INFORMATION NUTRITIONNELLES

(1) *Wylie-Rosett J*
Dairy products and metabolic risk factors: how much do we know?
Diabetes Care 2011;34:1064-1065

(2) *Fumeron F, Lamri A, Abi Khalil C* et al.
Dairy Consumption and the Incidence of Hyperglycemia and the Metabolic Syndrome: Results from a French prospective study, Data from the Epidemiological Study on the Insulin Resistance Syndrome (DESIR).
Diabetes Care 2011;34:813-817

(3) *Fumeron F, Lamri A, Emery N* et al.
Dairy products and the metabolic syndrome in a prospective study, DESIR.
J Am Coll Nutr 2011;30:454S-463S

(4) *Sluijs I, Forouhi NG, Beulens JW* et al.
The amount and type of dairy product intake and incident type 2 diabetes: results from the EPIC-InterAct Study.
Am J Clin Nutr 2012;96:382-390

(5) *Malik VS, Sun Q, van Dam RM* et al.
Adolescent dairy product consumption and risk of type 2 diabetes in middle-aged women.
Am J Clin Nutr 2011;94:854-861

(6) *Aune D, Norat T, Romundstad P, Vatten LJ*
Dairy products and the risk of type 2 diabetes: a systematic review and dose-response meta-analysis of cohort studies.
Am J Clin Nutr 2013;

(7) *Hodge AM, English DR, O'Dea K* et al.
Plasma phospholipid and dietary fatty acids as predictors of type 2 diabetes: interpreting the role of linoleic acid.
Am J Clin Nutr 2007;86:189-197

(8) *Krachler B, Norberg M, Eriksson JW* et al.
Fatty acid profile of the erythrocyte membrane preceding development of Type 2 diabetes mellitus.
Nutr Metab Cardiovasc Dis 2008;18:503-510

(9) *Mozaffarian D, Cao H, King IB* et al.
Trans-palmitoleic acid, metabolic risk factors, and new-onset diabetes in U.S. adults: a cohort study.
Ann Intern Med 2010;153:790-799

(10) *Mozaffarian D, de Oliveira Otto MC, Lemaitre RN* et al.
trans-Palmitoleic acid, other dairy fat biomarkers, and incident diabetes: the Multi-Ethnic Study of Atherosclerosis (MESA).
Am J Clin Nutr 2013;97:854-861

(11) *Panwar H, Rashmi HM, Batish VK, Grover S*
Probiotics as potential biotherapeutics in the management of type 2 diabetes - prospects and perspectives.
Diabetes Metab Res Rev 2013;29:103-112

(12) *Panagiotakos DB, Pitsavos CH, Zampelas AD* et al.
Dairy products consumption is associated with decreased levels of inflammatory markers related to cardiovascular disease in apparently healthy adults: the ATTICA study.
J Am Coll Nutr 2010;29:357-364

(13) *Rice BH, Quann EE, Miller GD*
Meeting and exceeding dairy recommendations: effects of dairy consumption on nutrient intakes and risk of chronic disease.
Nutr Rev 2013;71:209-223

(14) *Pittas AG, Dawson-Hughes B, Li T* et al.
Vitamin D and calcium intake in relation to type 2 diabetes in women.
Diabetes Care 2006;29:650-656

(15) *Liu S, Choi HK, Ford E* et al.
A prospective study of dairy intake and the risk of type 2 diabetes in women.
Diabetes Care 2006;29:1579-1584

Figure 1 : Méta-analyse : Risques relatifs de diabète de type 2 associés à la consommation de produits laitiers (pour 400g/jour). Test d'hétérogénéité entre études (P=0,13) d'après Aune *et al.*(6)

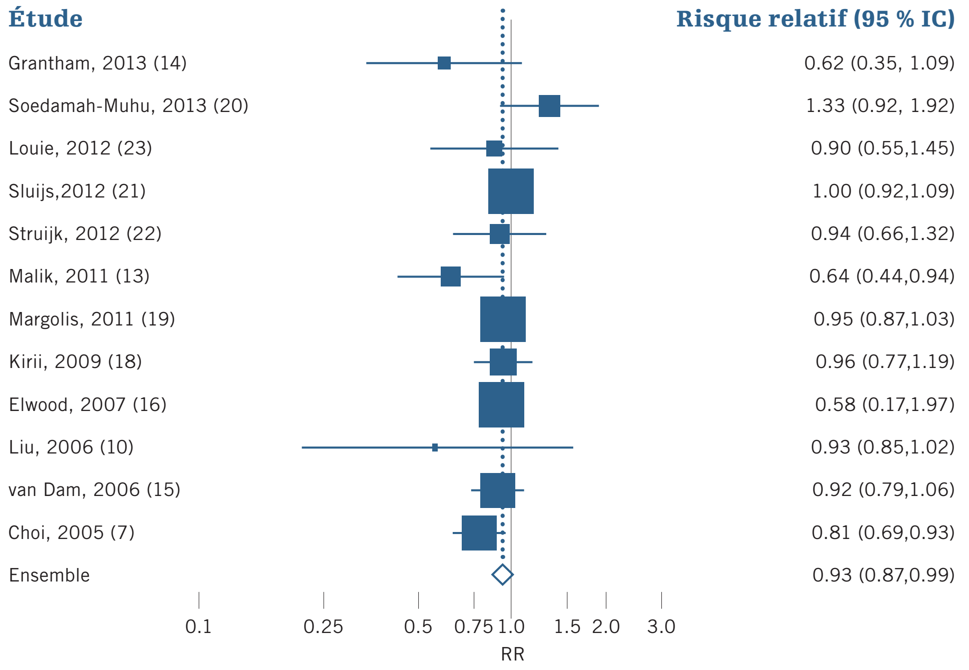

Tableau 2 : Risque relatifs de diabète de type 2 pour les consommations des différents types de produits laitiers (D'après la méta-analyse d'Aune *et al.* (6), 2013)

	Catégorie la plus haute *vs.* plus basse		Effet dose		
**Item**	**Nombre d'études**	**RR combiné (95% IC)**	**Nombre d'études**	**RR combiné (95% IC)**	**Pour une dose**
**Tous produits laitiers**	14	0,89 (0,82-0,96)	12	0,93 (0,87-0,99)	**400g/jour**
**Produits laitiers allégés**	10	0,83 (0,76-0,90)	9	0,91 (0,86-0,96)	**200g/jour**
**Produits laitiers entiers**	8	0,96 (0,87-1,06)	9	0,98 (0,94-1,03)	**200g/jour**
**Fromage**	8	0,91 (0,84-0,98)	8	0,92 (0,86-0,99)	**50g/jour**
**Lait***	7	0,87 (0,70-1,07)	7	0,87 (0,72-1,04)	**200g/jour**
**Yaourt***	7	0,86 (0,75-0,98)	7	0,78 (0,60-1,02)	**200g/jour**

RR = risque relatif, **IC** = intervalle de confiance. Lorsque la valeur 1,00 est comprise dans l'intervalle de confiance, la relation est non significative.
**l'analyse montre une hétérogénéité importante entre études pour le lait et le yaourt*

Tableau 3 : Relations entre biomarqueurs de la consommation de produits laitiers et incidence du diabète dans les études prospectives

Pays, Cohorte, durée du suivi, référence	Caractéristiques des sujets (n cas incidents/total)	Résultats
Australie, Melbourne Collaborative Cohort Study, 4 ans<br>*Hodge et al. 2007* (7)	Hommes et femmes, 36–72 ans<br>(364/3737)	L'acide gras C15:0 des phospholipides plasmatiques est négativement associé à l'incidence du diabète. Les diabétiques consomment moins de C15:0, mais cette consommation estimée n'est pas significativement associée à l'incidence après prise en compte des variables d'ajustement. ➔ imprécision des méthodes d'estimation des apports par rapport aux dosages…
Suède, Västerbotten Intervention Programme (étude nichée cas–témoins) 8,8 ans<br>*Krachler et al. 2008* (8)	Hommes et femmes, âge moyen 51,5 ans<br>(159/450)	Les acides gras C15:0 and C17:0 des membranes érythrocytaires sont négativement associés à l'incidence du diabète.
USA, Cardiovascular Health Study 14 ans<br>*Mozaffarian et al. 2010* (9)	Hommes et femmes<br>>65 ans<br>(304/2985)	L'acide transpalmitoléique des phospholipides du plasma est négativement associée à l'incidence du diabète (HR=0,41 (95% IC 0,27-0,64) et 0,38 (0,24-0,62) pour les quintiles 4 et 5 *vs.* quintile 1. La consommation de produits laitiers entiers, mais non allégés, est négativement associée à l'incidence du diabète, mais la relation n'est plus significative après ajustement sur le transpalmitoléate.
USA, Multi-Ethnic Study of Atherosclerosis, 7 ans<br>*Mozaffarian et al. 2013* (10)	Hommes et femmes, 45-84 ans<br>(205/2281)	L'acide transpalmitoléique des phospholipides du plasma quintile 5 *vs.* quintile 1 (HR: 0,52; 95% IC: 0,32-0,85).<br>La concentration de transpalmitoléate reste significativement associée après ajustement sur les différents produits laitiers.

Âge à l'inclusion. RR=risque relatif, OR=odds ratio, HR=hazard ratio, IC=intervalle de confiance.

à un ensemble de comportements « sains » et non directement à ces produits. Il faut noter que toutes ces études prennent en compte dans les analyses statistiques un grand nombre de variables d'environnement et de mode de vie, de manière à éliminer ce biais.

Les études de consommation sont difficiles à réaliser et comportent une large part d'imprécision. Elles reposent pour la plupart sur des auto-questionnaires qui peuvent être biaisés par une mémoire imparfaite ou sélective, ou des erreurs dans le report des différents types d'aliments (et de produits laitiers). La recherche de biomarqueurs spécifiques et dosables de manière objective pallie ces inconvénients. Ainsi, des études ont corrélé des marqueurs spécifiques des produits laitiers, comme les acides gras en C15 et C17 [7; 8], ou l'acide transpalmitoléique [9; 10], avec l'incidence du diabète. Ces études sont résumées dans le **tableau 3**. Dans l'ensemble, ces marqueurs sont négativement associés au risque. La nature lipidique de ces marqueurs montre que même les matières grasses laitières pourraient jouer un rôle protecteur.

Hypothèses de mécanisme

Le lait et ses produits dérivés contiennent de nombreux nutriments pouvant être à l'origine de la relation avec le diabète de type 2 : calcium et autres minéraux, vitamine D, protéines et peptides, acides gras. Les effets bénéfiques sur le diabète de type 2 observés avec les produits laitiers, en particulier avec les produits fermentés, pourraient également provenir des probiotiques qu'ils contiennent [11]. L'importance du microbiote dans l'obésité et les troubles métaboliques est mise en évidence depuis quelques années et a été développée récemment dans **Cholédoc (n°129)**. Nous n'insisterons donc pas sur cet aspect dans cette revue.

Le diabète de type 2 résulte de la conjonction d'une résistance à l'insuline et d'un déficit d'insulino-sécrétion. Les nutriments du lait jouent sur l'une et/ou l'autre de ces composantes. En particulier, l'inflammation, entraînant la résistance à l'insuline, est souvent un des mécanismes en cause. Dans l'étude ATTICA, la consommation de produits laitiers est inversement associée chez des adultes en bonne santé, aux marqueurs de l'inflammation IL-6, TNFα, et CRP, avec un effet dose [12]. Dans plusieurs essais cliniques (études d'intervention), la consommation de divers types de produits laitiers est associée à une baisse des marqueurs de l'inflammation [revue dans 13].

Calcium, minéraux et vitamines

Dans l'étude des infirmières américaines [14], les femmes consommant plus de trois portions de produits laitiers avaient une diminution du risque de diabète de type 2 de 11% par rapport à celles consommant moins d'une portion par jour. L'ajustement sur le calcium et la vitamine D réduisait notablement cette association, montrant que l'effet des produits laitiers était dû en grande partie à ces nutriments. Cependant dans plusieurs études, l'ajustement sur les minéraux et/ou la vitamine D ne modifie pas le résultat [15; 16]. Dans DESIR, l'effet de la teneur calcique est important, mais on est dans l'impossibilité d'ajuster la consommation de produits laitiers pour le calcium puisque sa consommation a été justement calculée d'après les produits laitiers, et non mesurée. D'autre part, nous n'avons pas de données concernant la vitamine D. Cependant il est possible que cet effet vitamine D soit plus important aux USA puisque les produits laitiers y sont très largement enrichis. La vitamine D pourrait influencer la sécrétion d'insuline et réduire l'insulino-résistance.

Le calcium augmente la sécrétion d'insuline et est indispensable aux tissus répondeurs à l'insuline comme le muscle squelettique et le tissu adipeux. Le calcium alimentaire régule négativement le calcium intra-cellulaire [17-19] ce qui entraîne dans les adipocytes une stimulation de la lipolyse et une inhibition de la lipogénèse. Le calcium pourrait modifier également le métabolisme lipidique par inhibition de l'absorption intestinale. De nombreuses expériences sur modèles animaux montrent ainsi un effet sur la corpulence. Chez l'homme, la consommation de calcium est parfois associée négativement à l'IMC mais pas toujours. De plus, dans la plupart des études rapportées ici, un ajustement sur l'IMC a été effectué, ce qui montre que si l'effet des produits laitiers sur le risque de diabète de type 2 passe par le calcium, c'est indépendamment d'un effet sur la corpulence.

D'autres minéraux sont fournis également par les produits laitiers, comme le magnésium et le potassium qui semblent avoir une action protectrice vis-à-vis du diabète de type 2 et de la résistance à l'insuline [20; 21]. Il faut cependant souligner que les essais de supplémentation en minéraux apportent peu de bénéfices, comme le souligne l'éditorial de Diabetes Care sur nos résultats dans l'étude DESIR [1].

Le fromage et les produits laitiers sont également une source de vitamines K, qui ont été également associées au diabète de type 2 [22]. Cette influence pourrait s'exercer par un effet anti-inflammatoire [23].

Protéines

Les protéines du lactosérum possèdent des propriétés anti-obésité et anti-diabète de type 2 par différents mécanismes biochimiques et métaboliques [24]. Les protéines du lait particulièrement

(16) *Margolis KL, Wei F, de Boer IH* et al.
A diet high in low-fat dairy products lowers diabetes risk in postmenopausal women.
J Nutr 2011;141:1969-1974

(17) *Zemel MB*
Mechanisms of dairy modulation of adiposity.
J Nutr 2003;133:252S-256S

(18) *Zemel MB, Sun X*
Dietary calcium and dairy products modulate oxidative and inflammatory stress in mice and humans.
J Nutr 2008;138:1047-1052

(19) *van Meijl LE, Vrolix R, Mensink RP*
Dairy product consumption and the metabolic syndrome.
Nutr Res Rev 2008;21:148-157

(20) *Ma B, Lawson AB, Liese AD* et al.
Dairy, magnesium, and calcium intake in relation to insulin sensitivity: approaches to modeling a dose-dependent association.
Am J Epidemiol 2006;164:449-458

(21) *Heianza Y, Hara S, Arase Y* et al.
Low serum potassium levels and risk of type 2 diabetes: the Toranomon Hospital Health Management Center Study 1 (TOPICS 1).
Diabetologia 2011;54:762-766

(22) *Beulens JW, van der AD, Grobbee DE* et al.
Dietary phylloquinone and menaquinones intakes and risk of type 2 diabetes.
Diabetes Care 2010;33:1699-1705

(23) *Shea MK, Dallal GE, Dawson-Hughes B* et al.
Circulating cytokines, and bone mineral density in older men and women.
Am J Clin Nutr 2008;88:356-363

(24) *Jakubowicz D, Froy O*
Biochemical and metabolic mechanisms by which dietary whey protein may combat obesity and Type 2 diabetes.
J Nutr Biochem 2013;24:1-5

(25) *Ma J, Stevens JE, Cukier K* et al.
Effects of a protein preload on gastric emptying, glycemia, and gut hormones after a carbohydrate meal in diet-controlled type 2 diabetes.
Diabetes Care 2009;32:1600-1602

(26) *Tulipano G, Sibilia V, Caroli AM, Cocchi D*
Whey proteins as source of dipeptidyl dipeptidase IV (dipeptidyl peptidase-4) inhibitors.
Peptides 2011;32:835-838

(27) *Pfeuffer M, Schrezenmeir J*
Milk and the metabolic syndrome.
Obes Rev 2007;8:109-118

(28) *Zhou J, Keenan MJ, Losso JN* et al.
Dietary whey protein decreases food intake and body fat in rats.
Obesity (Silver Spring) 2011;19:1568-1573

(29) *Brown JM, McIntosh MK*
Conjugated linoleic acid in humans: regulation of adiposity and insulin sensitivity.
J Nutr 2003;133:3041-3046

(30) *Moloney F, Toomey S, Noone E* et al.
Antidiabetic effects of cis-9, trans-11-conjugated linoleic acid may be mediated via anti-inflammatory effects in white adipose tissue.
Diabetes 2007;56:574-582

(31) *Field CJ, Blewett HH, Proctor S, Vine D*
Human health benefits of vaccenic acid.
Appl Physiol Nutr Metab 2009;34:979-991

(32) *Niu K, Kobayashi Y, Guan L et al.* Low-fat dairy, but not whole-/high-fat dairy, consumption is related with higher serum adiponectin levels in apparently healthy adults. *Eur J Nutr 2013;52:771-778*

(33) *Herranz D, Serrano M* SIRT1:recent lessons from mouse models. *Nat Rev Cancer 2010;10:819-823*

(34) *Bruckbauer A, Zemel MB* Effects of dairy consumption on SIRT1 and mitochondrial biogenesis in adipocytes and muscle cells. *Nutr Metab (Lond) 2011;8:91*

(35) *Choi HK, Willett WC, Stampfer MJ et al.* Dairy consumption and risk of type 2 diabetes mellitus in men: a prospective study. *Arch Intern Med 2005;165:997-1003*

(36) *Montonen J, Jarvinen R, Heliovaara M et al.* Food consumption and the incidence of type II diabetes mellitus. *Eur J Clin Nutr 2005;59:441-448*

(37) *van Dam RM, Hu FB, Rosenberg L et al.* Dietary calcium and magnesium, major food sources, and risk of type 2 diabetes in U.S. black women. *Diabetes Care 2006;29:2238-2243*

(38) *Lecomte P, Vol S, Caces E, Born C et al.* Five-year predictive factors of type 2 diabetes in men with impaired fasting glucose. *Diabetes Metab 2007;33:140-147*

(39) *Elwood PC, Pickering JE, Fehily AM* Milk and dairy consumption, diabetes and the metabolic syndrome: the Caerphilly prospective study. *J Epidemiol Community Health 2007;61:695-698*

(40) *Vang A, Singh PN, Lee JW et al.* Meats, processed meats, obesity, weight gain and occurrence of diabetes among adults: findings from Adventist Health Studies. *Ann Nutr Metab 2008;52:96-104*

(41) *Kirii K, Mizoue T, Iso H et al.* Calcium, vitamin D and dairy intake in relation to type 2 diabetes risk in a Japanese cohort. *Diabetologia 2009;52:2542-2550*

(42) *Villegas R, Gao YT, Dai Q et al.* Dietary calcium and magnesium intakes and the risk of type 2 diabetes: the Shanghai Women's Health Study. *Am J Clin Nutr 2009;89:1059-1067*

(43) *Soedamah-Muthu SS, Masset G, Verberne et al.* Consumption of dairy products and associations with incident diabetes, CHD and mortality in the Whitehall II study. *Br J Nutr 2012:jun7:1-9 [Epub ahead of print]*

(44) *Struijk EA, Heraclides A, Witte DR et al.* Dairy product intake in relation to glucose regulation indices and risk of type 2 diabetes. *Nutr Metab Cardiovasc 2013;23:822-28*

(45) *Louie JC, Flood VM, Rangan AM et al.* Higher regular fat dairy consumption is associated with lower incidence of metabolic syndrome but not type 2 diabetes. *Nutr Metab Cardiovasc 2013;23:816-21*

(46) *Grantham NM, Magliano DJ, Hodge A et al.* The association between dairy food intake and the incidence of diabetes in Australia: the Australian Diabetes Obesity and Lifestyle Study (AusDiab). *Public Health Nutr 2013;16:339-345*

celles du lactosérum, stimulent la sécrétion d'insuline. Ceci semble en contradiction avec l'amélioration de la résistance à l'insuline observée dans de nombreuses études dont DESIR [2; 3]. En réalité, les protéines du lactosérum élèvent l'insuline par un effet sur les incrétines, ce qui améliore la glycémie post-prandiale après un repas glucidique [25]. Les médicaments anti-diabétiques les plus récents sont justement des médicaments à effet incrétine. Ainsi, on a identifié dans la β lactoglobuline du lactosérum un tripeptide (Ile-Pro-Ala), aux effets inhibiteurs de l'enzyme DPP4, principe d'une partie de ces médicaments aux effets incrétine [26]. Administrées de manière chronique, après 12 semaines, les protéines du lactosérum diminuent l'insulinémie de 11% chez des sujets en surpoids ou obèses [27], ce qui montre une amélioration de la résistance à l'insuline. De plus, ces protéines diminuent la prise alimentaire par des effets sur les médiateurs peptidiques digestifs de la faim et la satiété, augmentent la thermogénèse, et réduisent la masse adipeuse dans les modèles animaux [24; 28].

Acides gras

Les acides gras conjugués de l'acide linoléique (CLA) ont été extensivement étudiés en relation avec l'obésité et la sensibilité à l'insuline [29]. Le CLA 9cis, 11*trans*, acide ruménique, majoritaire dans les produits laitiers, améliore la sensibilité à l'insuline par une augmentation de l'expression du récepteur nucléaire PPARγ. Chez les rats Zucker génétiquement obèses, les CLA diminuent l'insulinémie et la pression artérielle, et augmentent l'adiponectine. Les effets antidiabétiques du CLA 9c, 11t pourraient être liés à un effet anti-inflammatoire dans le tissu adipeux blanc. D'autres acides gras du lait pourraient aussi jouer un rôle [30]. Les acides gras trans produits à partir de l'hydrogénation partielle des huiles dans l'industrie agro-alimentaire augmentent le risque cardiovasculaire. Cependant, l'acide trans vaccénique (18:1 t11) est un précurseur du CLA 9c, 11t et est un agoniste de PPARα, comme les médicaments de la classe des fibrates, ce qui améliore le métabolisme lipidique [31].

Nous avons déjà mentionné plus haut que les acides gras saturés à nombre impair de carbone (C15:0 et C17:0) et l'acide transpalmitoléique (16:1 t7), fournis essentiellement par les produits laitiers, sont associés négativement au diabète de type 2 dans les études de cohorte [9]. Cependant, on ne sait pas s'ils ont des effets directs ou s'ils sont simplement des marqueurs de consommation.

Adiponectine, SIRT1

L'adiponectine est sécrétée par le tissu adipeux. Paradoxalement, sa concentration plasmatique est plus basse chez les obèses. Elle est insulin-sensibilisatrice et anti-inflammatoire. Dans une étude japonaise, la consommation de produits laitiers allégés est corrélée à une concentration augmentée, en analyse transversale et un an plus tard [32].

SIRT1, est une enzyme de la famille des sirtuines, dont un des rôles le mieux démontré est un effet protecteur contre l'intolérance au glucose et la stéatose hépatique induites par des régimes riches en graisse [33]. Le sérum de sujets en surpoids ou obèses ayant consommé des suppléments en produits laitiers pendant 28 jours entraîne une surexpression de SIRT1 dans des adipocytes et des cellules musculaires en culture [34]. Cette surexpression provoque, entre autres, une diminution des cytokines pro-inflammatoires IL-6 et TNF-α. Le calcium et certains acides aminés comme la leucine pourraient être les principaux composants responsables de ces effets.

Pour conclure

Un ensemble d'études prospectives est en faveur d'un effet protecteur des produits laitiers sur le risque de diabète de type 2. Cependant les effets des différents types de produits sont difficiles à établir de manière certaine. Les produits laitiers contiennent un mélange complexe de nutriments tous susceptibles de jouer un rôle sur l'association avec le risque de diabète de type 2. Les études épidémiologiques ne permettent pas de mettre en évidence de manière individuelle les effets de ces nutriments, d'autant plus que leur consommation est la plupart du temps estimée et non mesurée à partir des données de consommation des produits laitiers. L'intercorrélation de ces variables rend les calculs ajustés peu fiables ou impossibles à réaliser. De plus, il est très vraisemblable que ces différents composants agissent en interaction (effet « matrice »). Des dosages pour certains composants spécifiques comme certains acides gras confortent cependant l'association inverse entre produits laitiers et diabète de type 2 par des mesures «objectives ».

Frédéric Fumeron
Inserm U695,
UFR de médecine de l'Université Paris Diderot, Paris

Contacts: Dr M.-C. Bertière - Y. Soustre, Dr ès Sc.
Centre de Recherche et d'Information Nutritionnelles - 42 rue de Châteaudun- 75314 PARIS CEDEX 09
fax: 01 42 80 64 13 - Email: nutrition-fr@cerin.org

Maquette : la-fabrique-créative — ISSN 1639-2558

Tableau 1 : Relations entre consommation de produits laitiers et incidence du diabète dans les études prospectives

Pays, Cohorte, durée du suivi, référence	Caractéristiques des sujets (n cas incidents/total)	Résultats
USA, Health Professionals Follow-up Study, 12 ans<br>Choi et al. 2005 [35]	Hommes, 40–75 ans<br>(1243/41254)	Produits laitiers totaux inversement associés à l'incidence du diabète.<br>Produits laitiers allégés et yaourt inversement associés à l'incidence du diabète<br>Lait entier, ou produits laitiers entiers non associés à l'incidence du diabète.<br>Ajustement sur magnésium et potassium ne modifie pas les associations. L'ajustement sur le calcium réduit la signification statistique mais ne modifie pas l'ampleur de l'association.
Finlande, Finnish Mobile Clinic Health Examination Survey, 23 ans<br>*Montonen et al., 2005* [36]	Hommes et femmes, 40-69 ans<br>(383/4304)	Produits laitiers entiers non associés significativement<br>Produits allégés non associés<br>Lait entier non associé
USA, Women's Health Study, 10 ans<br>*Liu et al. 2006* [15]	Femmes, âge moyen 55 ans<br>(1603/37 183)	Produits laitiers totaux inversement associés à l'incidence du diabète.<br>Produits laitiers allégés, yaourt, inversement associés à l'incidence du diabète<br>Produits laitiers entiers non associés.<br>La relation persiste après ajustement sur le calcium, la vitamine D, le magnésium.
USA, Black Women's Health Study, 8 ans<br>*Van Dam et al. 2006* [37]	Femmes noires, 21-69 ans<br>(1964/41 186)	Produits laitiers totaux non associés<br>Produits entiers non associés<br>Produits allégés non significatifs mais tendance à la diminution
USA, Nurses' Health Study, 20 ans<br>*Pittas et al. 2006* [14]	Femmes, 35-55 ans<br>(4843/83779)	Produits laitiers totaux inversement associés à l'incidence<br>La consommation de calcium et de vitamine D est associée négativement au diabète de type 2.<br>L'ajustement sur la consommation de calcium et de vitamine D diminue l'association avec les produits laitiers :
France, sanstitre, 5 ans<br>*Lecomte et al. 2007* [38]	Hommes pré-diabétiques, 20-60 ans<br>(127/743)	L'absence de consommation journalière de laitages augmente le risque : OR 1,86 (1.21–2.86)
Royaume Uni, Caerphilly prospective study, 25 ans<br>*Elwood et al., 2007* [39]	Hommes, 45-59 ans<br>(41/640)	Produits laitiers totaux non associés
USA, Adventist Health Study, 17 ans<br>*Vang et al. 2008* [40]	Hommes et femmes, 45-88 ans<br>(543/8401)	Fromage et lait : non associés
Japon, Japan Public Health Center-based Prospective Study, 5 ans<br>*Kirii et al., 2009* [41]	Hommes, 40-69 ans<br>(634/25877)<br>Femmes, 40-69 ans<br>(480/33919)	Chez les hommes, produits laitiers totaux, lait, fromage, yaourt non associés.<br>Chez les femmes, produits laitiers totaux associés négativement à l'incidence du diabète de type 2. Pas d'association significative avec le lait, fromage, yaourts séparément.<br>Il existe une association négative avec la consommation de calcium chez les plus hauts consommateurs de vitamine D (hommes et femmes).
Chine, Shangai Women's Health Study, 6,9 ans<br>*Villegas et al., 2009* [42]	Femmes, âge moyen 51 ans<br>(2270/64191)	La consommation de lait est associée négativement au diabète de type 2. (>200g/jour vs. 0, RR= 0,60 (0,41–0,88))<br>Calcium quelle que soit la source (animal, laitière, végétale) et magnésium (animal, laitier) inversement associés.
USA, Nurses' Health Study II, 7 ans<br>*Malik et al. 2011* [5]	Femmes, 34–53 ans<br>(550/37038)	Consommation de produits entiers à l'adolescence associée inversement à l'incidence du diabète (quintile supérieur (≥2 portions/jour) : RR 0,62 (0,47-0,83). L'association disparait avec ajustement sur la consommation à l'âge adulte ce qui montre que l'effet est lié à la persistance du comportement à l'âge adulte.<br>Pas d'association avec produits allégés à l'adolescence.<br>Consommations à l'âge adulte de produits entiers et de produits allégés associées négativement à l'incidence du diabète.
France, Données épidémiologiques sur le syndrome d'insulino-résistance (D.E.S.I.R.), 9 ans<br>*Fumeron et al. 2011* [2; 3]	Hommes et femmes, 30–65 ans<br>(200/3435)	Produits laitiers sauf fromages associés négativement à la survenue d'une hyperglycémie et/ou d'un diabète de type 2 (OR= 0,85; 95%CI, 0,76–0,94). L'association avec le diabète de type 2 seul n'est plus significative après ajustement sur l'IMC.<br>Le fromage n'est pas associé à l'incidence du diabète de type 2.
USA, Women's Health Initiative Observational Study, 8 ans<br>*Margolis et al. 2011* [16]	Femmes, 50–79 ans<br>(3946/82076)	Produits laitiers allégés et yaourts inversement associés à l'incidence du diabète (5ème quintile vs. 1er quintile : RR=0,65 (0,44–0,96) et 0,46 (0,31-0,68 respectivement). Pas d'association avec les produits entiers. L'ajustement pour calcium, magnésium, et la vitamine D ne modifie pas les résultats.
EPIC-Interact (Royaume-Uni, France, Italie, Allemagne, Pays-Bas, Espagne, Danemark, Suède), suivi médian témoins 12,3 ans, cas : 6,8 ans<br>*Sluijs et al. 2012* [4]	Hommes et femmes, âge moyen 52 ans, (12403/340234 dont seuls 16835 tirés au sort ont été comparés aux cas diabétiques)	Comparaison du quintile 5 vs. quintile 1. ➔ Produits laitiers totaux et lait non associés. Fromage : HR=0,88; 95% CI: 0,76-1,02. Produits laitiers fermentés (fromage, yaourt, lait fermenté épais : HR=0,88; 95% IC: 0,78-0,99) .<br>L'ajustement sur les minéraux + vitamine D renforce le résultat montrant que l'association est sûrement liée à d'autres composants
Grande Bretagne, Whitehall II study, 10 ans<br>*Soedamah-Muthu et al. 2013* [43]	Hommes et femmes 35–55 ans<br>(273/4186)	Pas d'association des produits laitiers totaux, entiers ou allégés, lait, yaourt, fromage avec l'incidence du diabète
Danemark, Inter99 Study, 5 ans<br>*Struijk et al. 2012* [44]	Hommes et femmes, 30-60 ans<br>(214/5953)	Pas d'association des produits laitiers totaux, allégés, entiers, lait, fromage, produits fermentés à l'incidence du diabète<br>Mais fromage négativement associés à la glycémie 2h post HGPO, produits fermentés négativement associés à la glycémie à jeun et à HbA1c.
Australie, Blue Mountains Eye study, 10 ans<br>*Louie et al., 2012* [45]	Hommes et femmes, >49 ans<br>(155/1824)	Produits laitiers totaux, produits allégés, produits entiers non associés
Australie, The Australian Diabetes Obesity and Lifestyle Study, 5 ans<br>*Grantham et al. 2013* [46]	Hommes et Femmes, > 25 ans<br>(209/5582)	Produits laitiers totaux (3ème tertile vs. 1er tertile) négativement associés à l'incidence : OR 0,53 (IC 95% 0,29-0,96).

Âge à l'inclusion. RR=risque relatif, OR=odds ratio, HR=hazard ratio, IC=intervalle de confiance.

Une sélection bibliographique de CERINUT

sur l'actualité

Albers R, Bourdet-Sicard R, Braun D et al.
Monitoring immune modulation by nutrition in the general population: identifying and substantiating effects on human health
Br J Nutr 2013 ; 110(Suppl 2) : S1-S30.

Alheritiere A, Montois S, Galinski M et al.
Worldwide relation between the number of McDonald's restaurants and the prevalence of obesity
J Intern Med 2013 ; doi:10.1111/joim.12126

Aubry C, Kebir L.
Shortening food supply chains: A means for maintaining agriculture close to urban areas? The case of the French metropolitan area of Paris
Food Policy 2013 ; 41 : 85-93.

Ballard KD, Mah E, Guo Y et al.
Low-Fat Milk Ingestion Prevents Postprandial Hyperglycemia-Mediated Impairments in Vascular Endothelial Function in Obese Individuals with Metabolic Syndrome
J Nutr 2013 ; doi:10.3945/jn.113.179465

Bel S, Michels N, De Vriendt T et al.
Association between self-reported sleep duration and dietary quality in European adolescents
Br J Nutr 2013 ; 110(5) : 949-59.

Bel-Serrat S, Mouratidou T, Jimenez-Pavon D et al.
Is dairy consumption associated with low cardiovascular disease risk in European adolescents? Results from the HELENA Study
Pediatr Obes 2013 ; doi:10.1111/j.2047-6310.2013.00187.x

Bel-Serrat S, Mouratidou T, Santaliestra-Pasias AM et al.
Clustering of multiple lifestyle behaviours and its association to cardiovascular risk factors in children: the IDEFICS study
Eur J Clin Nutr 2013 ; 67(8) : 848-54.

Beltran-Sanchez H, Harhay MO, Harhay MM et al.
Prevalence and trends of Metabolic Syndrome in the adult US population, 1999-2010
J Am Coll Cardiol 2013 ; 62(8) : 697-703.

Bernard JY, De Agostini M, Forhan A et al.
The Dietary n6:n3 Fatty Acid Ratio during Pregnancy Is Inversely Associated with Child Neurodevelopment in the EDEN Mother-Child Cohort
J Nutr 2013 ; 143(9) : 1481-8.

Bloch Eidner M, Qvistgaard Lund AS, Harboe BS et al.
Calories and portion sizes in recipes throughout 100 years: An overlooked factor in the development of overweight and obesity?
Scand J Public Health 2013 ; doi:10.1177/1403494813498468

Braegger C, Campoy C, Colomb VJ et al.
Vitamin D in the healthy European paediatric population
J Pediatr Gastroenterol Nutr 2013 ; 56(6) : 692-701.

Cahill LE, Chiuve SE, Mekary RA et al.
Prospective Study of Breakfast Eating and Incident Coronary Heart Disease in a Cohort of Male US Health Professionals
Circulation 2013 ; 128(4) : 337-343.

Clément C, Molines L, Chaix F et al.
Suivi post-gastrectomie longitudinale chez l'obèse: les besoins nutritionnels sont loin d'être couverts pendant la première année.
Information Diététique 2013 ; (2) : 20-9.

Cockburn E, Bell PG, Stevenson E.
Effect of milk on team sport performance after exercise-induced muscle damage
Med Sci Sports Exerc 2013 ; 45(8) : 1585-92.

Conklin AI, Maguire ER, Monsivais P.
Economic determinants of diet in older adults: systematic review
J Epidemiol Community Health 2013 ; 67(9):721-7.

Curry A.
Archaeology: The milk revolution
Nature 2013 ; 500(7460) : 20-22.

De Vos WM, Nieuwdorp M.
Genomics: A gut prediction
Nature 2013 ; 498(7452) : 48-9.

Dyer AA, Gupta R.
Epidemiology of childhood food allergy
Pediatr Ann 2013 ; 42(6) : 91-95.

Hanks AS, Just DR, Wansink B.
Preordering school lunch encourages better food choices by children
JAMA Pediatr 2013 ; 167(7) : 673-674.

Iuliano S, Woods J, Robbins J.
Consuming two additional serves of dairy food a day significantly improves energy and nutrient intakes in ambulatory aged care residents: a feasibility study
J Nutr Health Aging 2013 ; 17(6) : 509-13.

Just DR, Wansink B.
One Man's Tall Is Another Man's Small: How the Framing of Portion Size Influences Food Choice
Health Econ 2013 ; doi:10.1002/hec.2949

Kanda A, Nakayama K, Fukasawa T et al.
Post-exercise whey protein hydrolysate supplementation induces a greater increase in muscle protein synthesis than its constituent amino acid content
Br J Nutr 2013 ; 110(6) : 981-7.

Karlsson FH, Tremaroli V, Nookaew I et al.
Gut metagenome in European women with normal, impaired and diabetic glucose control
Nature 2013 ; 498(7452) : 99-103.

Kennedy ET, Luo H, Houser RF.
Dietary Supplement Use Pattern of U.S. Adult Population in the 2007-2008 National Health and Nutrition Examination Survey (NHANES)
Ecol Food Nutr 2013 ; 52(1) : 76-84.

Kim SH, Kim WK, Kang MH.
Effect of milk and milk products consumption on physical growth and bone mineral density in Korean adolescents
Nutr Res Pract 2013 ; 7(4) : 309-14.

Levitt M, Wilt T, Shaukat A.
Clinical implications of lactose malabsorption versus lactose intolerance
J Clin Gastroenterol 2013 ; 47(6) : 471-80.

Mccarron DA, Kazaks AG, Geerling JC et al.
Normal Range of Human Dietary Sodium Intake: A Perspective Based on 24-Hour Urinary Sodium Excretion Worldwide
Am J Hypertens 2013 ; [Epub ahead of print]

Mensink GB, Fletcher R, Gurinovic M et al.
Mapping low intake of micronutrients across Europe
Br J Nutr 2013 ; 110(4) : 755-73.

Odegaard AO, Jacobs DR Jr, Steffen LM et al.
Breakfast Frequency and Development of Metabolic Risk
Diabetes Care 2013 ; doi:10.2337/dc13-0316

Radavelli-Bagatini S, Zhu K, Lewis JR et al.
Association of Dairy Intake with Body Composition and Physical Function in Older Community-Dwelling Women
J Acad Nutr Diet 2013 ; Jul 19 : doi: 10.1016/j.jand.2013.05.019.

Romeu M, Aranda N, Giralt M et al.
Diet, iron biomarkers and oxidative stress in a representative sample of Mediterranean population
Nutr J 2013 ; 12(1) : 102. [Epub ahead of print]

Sanders TA, Lewis FJ, Goff LM et al.
SFAs do not impair endothelial function and arterial stiffness
Am J Clin Nutr 2013 ; 98(3) : 677-83.

Sette S, Le Donne C, Piccinelli R et al.
The third National Food Consumption Survey, INRAN-SCAI 2005-06: major dietary sources of nutrients in Italy
Int J Food Sci Nutr 2013 ; doi:10.3109/09637486.2013.816937

Shiby VK, Mishra HN.
Fermented milks and milk products as functional foods-a review
Crit Rev Food Sci Nutr 2013 ; 53(5) : 482-496.

Sondermeijer BM, Rana JS, Arsenault BJ et al.
Non-HDL cholesterol versus Apo B for risk of coronary heart disease risk in healthy individuals: the EPIC-Norfolk prospective population study
Eur J Clin Invest 2013 ; doi:10.1111/eci.12129

Tielemans SM, Altorf-van der Kuil W, Engberink MF et al.
Intake of total protein, plant protein and animal protein in relation to blood pressure: a meta-analysis of observational and intervention studies
J Hum Hypertens 2013 ; 27(9) : 564-571.

Traill WB, Mazzocchi M, Niedzwiedzka B et al.
The EATWELL project: Recommendations for healthy eating policy interventions across Europe
Nutr Bull 2013 ; 38(3) : 352-7.

Vasson MP.
Les compléments alimentaires: bénéfice/risque.
Information Diététique 2013 ; (2) : 11-6.

CERINUT regroupe près de 40 000 références bibliographiques d'articles sélectionnés dans la presse scientifique française ou étrangère (plus de 100 titres).
Des profils bibliographiques personnalisés peuvent vous être adressés gracieusement sur simple demande.
